# Ecological scenario and *Trypanosoma cruzi* DTU characterization of a fatal acute Chagas disease case transmitted orally (Espírito Santo state, Brazil)

**DOI:** 10.1186/s13071-016-1754-4

**Published:** 2016-08-31

**Authors:** Maria Augusta Dario, Marina Silva Rodrigues, Juliana Helena da Silva Barros, Samanta Cristina das Chagas Xavier, Paulo Sérgio D’Andrea, André Luiz Rodrigues Roque, Ana Maria Jansen

**Affiliations:** 1 Laboratory of Trypanosomatid Biology, Oswaldo Cruz Institute, Fiocruz, Rio de Janeiro, Rio de Janeiro Brazil; 2 Laboratory of Biology and Parasitology of Wild Reservoir Mammals, Oswaldo Cruz Institute, Fiocruz, Rio de Janeiro, Rio de Janeiro Brazil

**Keywords:** Mixed infections, *Trypanosoma cruzi* DTU, *Trypanosoma dionisii*, Triatomine, Oral infection, Acute chagas disease

## Abstract

**Background:**

*Trypanosoma cruzi* infection via oral route results in outbreaks or cases of acute Chagas disease (ACD) in different Brazilian regions and poses a novel epidemiological scenario. In the Espírito Santo state (southeastern Brazil), a fatal case of a patient with ACD led us to investigate the enzootic scenario to avoid the development of new cases. At the studied locality, *Triatoma vitticeps* exhibited high *T. cruzi* infection rates and frequently invaded residences.

**Methods:**

Sylvatic and domestic mammals in the Rio da Prata locality, where the ACD case occurred, and in four surrounding areas (Baia Nova, Buenos Aires, Santa Rita and Todos os Santos) were examined and underwent parasitological and serological tests. Triatomines were collected for a fecal material exam, culturing and mini-exon gene molecular characterization, followed by RFLP-PCR of H3/Alul. Paraffin-embedded cardiac tissue of a patient was washed with xylene to remove paraffin and DNA was extracted using the phenol-chloroform method. For genotype characterization, PCR was performed to amplify the 1f8, GPI and 18S rRNA genes. In the case of V7V8 SSU rRNA, the PCR products were molecularly cloned. PCR products were sequenced and compared to sequences in GenBank. Phylogenetic analysis using maximum likelihood method with 1000 bootstrap replicates was performed.

**Results:**

None of the animals showed positive hemocultures. Three rodents and two dogs showed signs of infection, as inferred from borderline serological titers. *T. vitticeps* was the only triatomine species identified and showed *T. cruzi* infection by DTUs TcI and TcIV. The analysis of cardiac tissue DNA showed mixed infection by *T. cruzi* (DTUs I, II, III and IV) and *Trypanosoma dionisii*.

**Conclusions:**

Each case or outbreak of ACD should be analyzed as a particular epidemiological occurrence. The results indicated that mixed infections in humans may play a role in pathogenicity and may be more common than is currently recognized. Direct molecular characterization from biological samples is essential because this procedure avoids parasite selection. *T. dionisii* may under certain and unknown circumstances infect humans. The distribution of *T. cruzi* DTUS TcIII and TcIV in Brazilian biomes is broader than has been assumed to date.

**Electronic supplementary material:**

The online version of this article (doi:10.1186/s13071-016-1754-4) contains supplementary material, which is available to authorized users.

## Background

The genus *Trypanosoma* (Trypanosomatidae, Kinetoplastida), which includes the subgenus *Schizotrypanum*, is composed of numerous species that are distributed worldwide. Humans or other mammals can serve as suitable hosts. With the exception of *Trypanosoma cruzi*, other species of this subgenus are restricted to bats. Due to their morphological similarity, these other species have been classically described as *T. cruzi*-like [1, 2]. The biological cycles of *Schizotrypanum* trypanosomes are similar, differing only in the identity of their mammalian hosts and their hemipteran vectors. *Trypanosoma cruzi marinkellei* is transmitted by triatomine insects of the genus *Cavernicola*, and *Trypanosoma dionisii* is transmitted by Cimicidae. Species of *Schizotrypanum* are the only trypanosomes described thus far that infect mammalian cells and multiply inside them as amastigotes [2–4]. There is still much to study regarding *T. dionisii* and *T. c. marinkellei*. Furthermore, despite being the subject of intensive study for more than 100 years, there are still several unanswered questions pertaining to the biology of *T. cruzi*.

Trypanosomiasis by *T. cruzi* is primarily a sylvatic enzooty. This flagellate species is widely distributed in nature, occurring from the southern United States (USA) through southern Argentina and Chile [5]. *Trypanosoma cruzi* circulates among 150 mammal species and is capable of colonizing almost any tissue of its mammalian hosts. It can also be transmitted by dozens of triatomine species [6]. The parasite transmission cycle is complex in nature because, in addition to its tremendous host species diversity, *T. cruzi* is highly genetically diverse [7]. Currently, six Discrete Typing Units (DTUs), TcI to TcVI, in addition to TcBat, are recognized [8–10]. Correlations among DTUs/geographical distribution/host species and pathogenicity are still controversial. Classically, TcII, TcV and TcVI were related to severe human diseases and TcI, TcIII and TcIV were related to the sylvatic cycle [10], but in the Amazon region, Colombia and Venezuela, reports have described human disease by TcI, TcIII and TcIV [11–16]. Although diverse studies have proposed these and other correlations, this topic still requires further clarification. *Trypanosoma cruzi* populations can be selected when they are grown under laboratory conditions or even when natural infections lead to erroneous conclusions regarding DTU variety and putative associations [17, 18]. Similarly, due to the undersampling of hosts and habitats, the ecology of the DTUs of *T. cruzi* is far from well understood.

In Brazil, the efficient control of Chagas disease (CD) due to intra-domiciliary transmission of *T. cruzi* by *Triatoma infestans* has been largely achieved. However, human infection by *T. cruzi* is re-emerging as a food-borne disease in previously non-endemic areas, such as the Amazon region, where it is associated with the ingestion of Açai juice [19–21]. The oral route transmission has been demonstrated to be a highly efficient mechanism of infection [22, 23]. Acute Chagas disease (ACD) cases and outbreaks involving triatomines, which were not previously considered as the main vector species for the contaminative vectorial route, demonstrate that any triatomine can act as a vector when it is related to oral transmission [24]. Moreover, in sylvatic environments, this mechanism is likely the primary means of infection between animals [25].

In the Espírito Santo State (ES), the invasion of domiciles by infected triatomines (mainly *Triatoma vitticeps* but also *Panstrongylus geniculatus*) is frequently reported in rural areas, primarily in mountainous regions that have irregular terrain [26]. *Triatoma vitticeps* is the more prevalent species in ES and can be found in Rio de Janeiro, Minas Gerais and Bahia states [27, 28]. *Triatoma vitticeps* occasionally forms colonies associated with opossum nests in peridomiciles and has high infection rates by flagellates, such as *T. cruzi* [29, 30]. In a study conducted between 2010 and 2012 (Dario, unpublished data), 55 *T. cruzi* isolates derived from *T. vitticeps* and *P. geniculatus* collected in ES subjected to molecular characterization, demonstrated the transmission of four *T. cruzi* DTUs (TcI, TcII, TcIII and TcIV).

Despite the high *T. cruzi* infection rates, *T. vitticeps* has always been considered a secondary vector of CD due to the long time interval between feeding and defecation, reducing the success for the classical triatomine-human route transmission [31]. Since 2007, according to the Espírito Santo state Health Department (Sesa/ES), only three cases of CD were reported in ES. The last case, in 2012, led to the death of a 2-year-old patient, and epidemiological investigation showed the cause to be ACD acquired by the oral route due to the manipulation of a recently dead (and infected) *T. vitticeps*.

This study had the following objectives: (i) to determine the *T. cruzi* DTU that was related to the fatal case that occurred in ES and (ii) to study the ecology of the transmission cycle of *T. cruzi* in the area where the ACD occurred and in nearby locations where triatomines continuously invade residences. The aim of these objectives is to contribute to the development of local control measures to prevent additional cases of CD.

## Methods

### Cardiac tissue sample

A cardiac fragment was collected during the *post mortem* patient’s exam, embedded in paraffin and sent to Sesa/ES. The cardiac fragment was kindly donated by Dr Janaina A. Shineider Casotti from Sesa/ES.

### Study area

The Guarapari municipality is located in the Central coast of the ES state. It is 594,487 km^2^ in size and has a population of 105,286; of these, 4758 live in rural areas. The rural area in which the case occurred (and where we examined the domestic and wild animals) is located mostly in mountainous areas, which contain remnants of Atlantic rainforest. The residents of the rural area report banana and coffee agriculture to be the main source of their income.

**Fig. 1:**
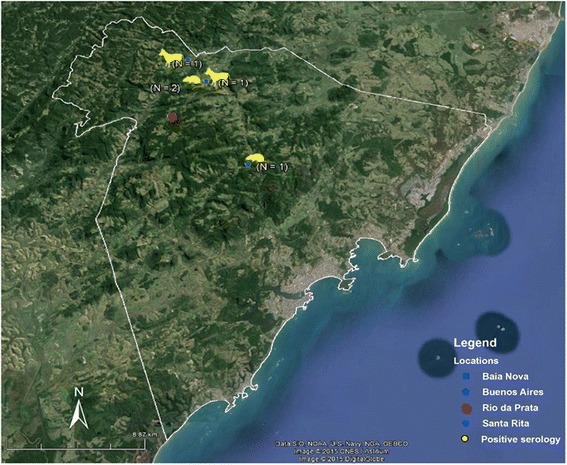
Representative map of the locality of the serological results of small sylvatic and domestic mammals. The positive mammals are framed in yellow. Only one rodent was positive at the Buenos Aires location and two were positive at the Baia Nova location. Two dogs were positive at the Baia Nova (n = 1) and Santa Rita (n = 1) locations

The study was conducted at five localities: Rio da Prata, where the patient's case occurred; Baia Nova; Buenos Aires; Todos os Santos; and Santa Rita (Fig. 1), which, according to Zoonosis Control Center (ZCC), have registered a higher number of infected triatomines invading residences in recent years.

### Small wild mammal capture

Fieldwork was conducted in June 2012, just a few months after the occurrence of the fatal case. Small wild mammals were captured using the following protocol: linear transects were designed in which capture points were established (each point 10 m apart) using alternating Shermann® (H. B. Sherman Traps, Tallahassee, FL, USA) and Tomahawk® (Tomahawk Live Traps, Tomahawk, WI, USA) live traps baited with a mixture of banana, peanut butter, bacon and sardine. The traps were placed near houses and in wild habitats. Seven transects with 10 Sherman and 10 Tomahawk traps each were placed in the field for five nights, with a total capture effort of 840 traps-night.

All captured animals were manipulated according to the safety manual for the use of wild mammals in research [32] and were anesthetized (9:1 ketamine chlorhydrate 10 % and acepromazine 2 %) for blood sample collection (by cardiac puncture) for parasitological and serological analyses. Only those mammals that required taxonomical confirmation obtained by karyotyping were sacrificed [33].

### Dog survey

An active search for dogs was performed at the following locations: Rio da Prata, Baia Nova and Santa Rita. At all of these locations, with the owner’s consent, a blood sample was collected by puncturing the cephalic vein under aseptic conditions with Vacutainer® tubes with anticoagulant for serological and parasitological tests. A canine questionnaire was given to the owner that requested the following information: name of the dog, age, sex and the dog’s primary function (protection, hunting or company). We considered dogs to be juveniles when they were less than one year of age and adults when they were more than one year of age. Each dog was considered a single event, even if they lived at the same house.

### *Trypanosoma cruzi* survey

To survey for *T. cruzi*, parasitological and serological tests were performed for both small wild mammals and dogs. The following parasitological tests were conducted: (i) fresh blood examination to visualize *T. cruzi* flagellates and (ii) hemoculture, which involved the inoculation of 0.6 ml of blood into two tubes containing Novy Mc Neal Nicole (NNN) medium with Liver Infusion Tryptose (LIT) overlay. The tubes were examined every two weeks for a total of three (for seronegative animals) or five months (for seropositive animals). When positive, the parasites were amplified in LIT, cryopreserved and deposited in the Coleção de Trypanosoma de Mamíferos Silvestres, Domésticos e Vetores, Fiocruz - COLTRYP (Oswaldo Cruz Foundation, Rio de Janeiro - RJ/Brazil). Positive hemoculture results also showed that the animal exhibited notable parasitemia levels.

A serological survey for the detection of anti-*T. cruzi* IgG antibodies was performed using an Indirect Immunofluorescent Antibody Test (IFAT), as described by [34]. The antigens used in the reaction were an equal mixture of parasites derived from the strains I00/BR/00 F (TcI) and MHOM/BR/1957/Y (TcII). The sera of Murinae rodents were tested with anti-rat IgG, while the sera of dogs were tested with anti-dog IgG. All sera were conjugated to fluorescein isothiocyanate (Sigma, St Louis, MO, USA). Echimyidae rodents and marsupials sera were tested using intermediary anti-IgG antibodies for *Thrichomys* spp. and anti-IgG for Didelphidae, respectively, both of which were raised in rabbits. The reaction was revealed using anti-rabbit IgG antibodies conjugated with fluorescein (Sigma, St Louis, MO, USA). The cutoff values for serological results were 1:40 for dogs and marsupials and 1:10 for rodents [35].

To avoid possible cross-reactions with other trypanosomatids, small mammals and dogs were screened to detect anti-*Leishmania* IgG antibodies through IFAT, as described above, using antigens derived from a mixture of *Leishmania infantum* and *L. braziliensis*. Animals were considered positive for *Leishmania* spp. when the serological titers for this parasite were higher than for *T. cruzi* by at least two dilutions and were considered to present both infections when titers were > 1:40 in each assay. Animals were considered to present *T. cruzi* infection when the serological titer was higher that the cutoff value analysis and/or when hemoculture were positive.

In-house Enzyme-Linked Immunosorbent Assays (ELISA) were performed to confirm infections in dogs by *T. cruzi* and *Leishmania* sp. The mean negative control optical density, which added 20 % to this value via a dog serum panel, established the cutoff values in each plate. For each serological reaction, two *T. cruzi* and *Leishmania* sp. positive and negative control sera were added.

### *Trypanosoma cruzi* survey in triatomines

Triatomines were collected inside houses by residents and delivered to the ZCC. Triatomine identification was performed according to the method of Lent & Wygodzinsky [36]. The intestinal contents were removed using scissors and forceps, and examined on a microscope slide with a cover slip under an optical microscope to search for flagellar forms similar to *T. cruzi*. When the exam was positive, the sample was cultured in NNN with LIT overlay and was examined every two weeks for up to five months [37, 38]. In addition, the culture was amplified, cryopreserved and deposited in COLTRYP, as described previously.

### *Trypanosoma cruzi* molecular characterization

#### DNA extraction

*T. cruzi* DNA derived from epimastigotes in the logarithmic phase of the cultures and DNA from the cardiac tissue embedded in the paraffin was extracted using the standard phenol-chloroform method [39]. Prior to this step, the cultures were washed with phosphate-buffered saline (PBS) solution and incubated with proteinase K (100 μg/ml) and 0.5 % sodium dodecyl sulfate (SDS) at 56 °C for two hours. For paraffin removal, the cardiac tissue was washed using a previously described method [40, 41]. DNA from the cardiac tissue was quantified using a NanoDrop 1000 Spectrophotometer (Thermo Scientific, San Jose, CA, USA), and the final concentration was adjusted to 50, 100, 150, 200 and 250 ng/μl. To avoid contamination, only unused aerosol-resistant pipette tips were used, and PCR was conducted in a separate room free of any *T. cruzi* or *T. dionisii* DNA (we do not have *T. dionisii* isolates in our laboratory). Irradiation with ultraviolet (UV) light was also performed on all materials, such as pipets, filter tips, PCR tubes and the cabinet area where the PCR was carried out.

#### Culture characterization

The parasite characterization of epimastigotes from positive cultures was performed as follows. First, multiplex-PCR was performed to amplify the non-transcribed spacer of the mini-exon gene [42] for the identification of TcI (DTU I), TcII (DTU II/V/VI), zymodeme 3 (DTU III/IV) and *T. rangeli* fragments of 200 bp, 250 bp, 150 bp and 100 bp [43], respectively, as well as mixed infections. Positive samples, except for TcI, were amplified by PCR for the histone 3 (H3) gene [44] followed by restriction fragment length polymorphism (RFLP) analysis. The fragments were digested by the AluI enzyme for discrimination of Z3 (DTUs III or IV).

Electrophoresis of PCR products was carried out in a 2 % agarose gel, which was stained with ethidium bromide solution and visualized under UV light. All reactions included distilled water as a negative control. *Trypanosoma cruzi* strains, representing all DTUs (TcI-SylvioX/10cl1; TcII-Esmeraldocl3; TcIII-M5631cl5; TcIV-92122102R and TcV/VI-SC43cl1), and *T. rangeli* (Choco) samples were used as positive controls.

#### Cardiac tissue characterization

For DTU identification, DNA extracted from cardiac tissue was used to amplify three nuclear markers: 1f8 (950 bp), glucose-phosphate isomerase (GPI) (652 bp) and the third portion of variable regions 7 and 8 (V7V8) of 18S rRNA gene (650 bp) (45), according to previous studies [46–48]. PCR products were purified using the Illustra GFX PCR DNA and gel band purification kit (GE Healthcare Life Sciences, Little Chalfont, Buckinghamshire, UK). In addition, the V7V8 region of SSU rRNA (750–800bp) was amplified as described [49].

V7V8 SSU rRNA PCR products were purified with a Wizard Genomic DNA Purification kit (Promega, Madison, WI, USA) and cloned using the pGEM-T Easy Vector System (Promega, Madison, WI, USA) per the manufacturer’s protocol. Sixteen colonies were randomly collected and minipreps were performed with Invisorb Spin Plasmid Mini Two kits (STRATEC Biomedical AG, Germany).

**Fig. 2:**
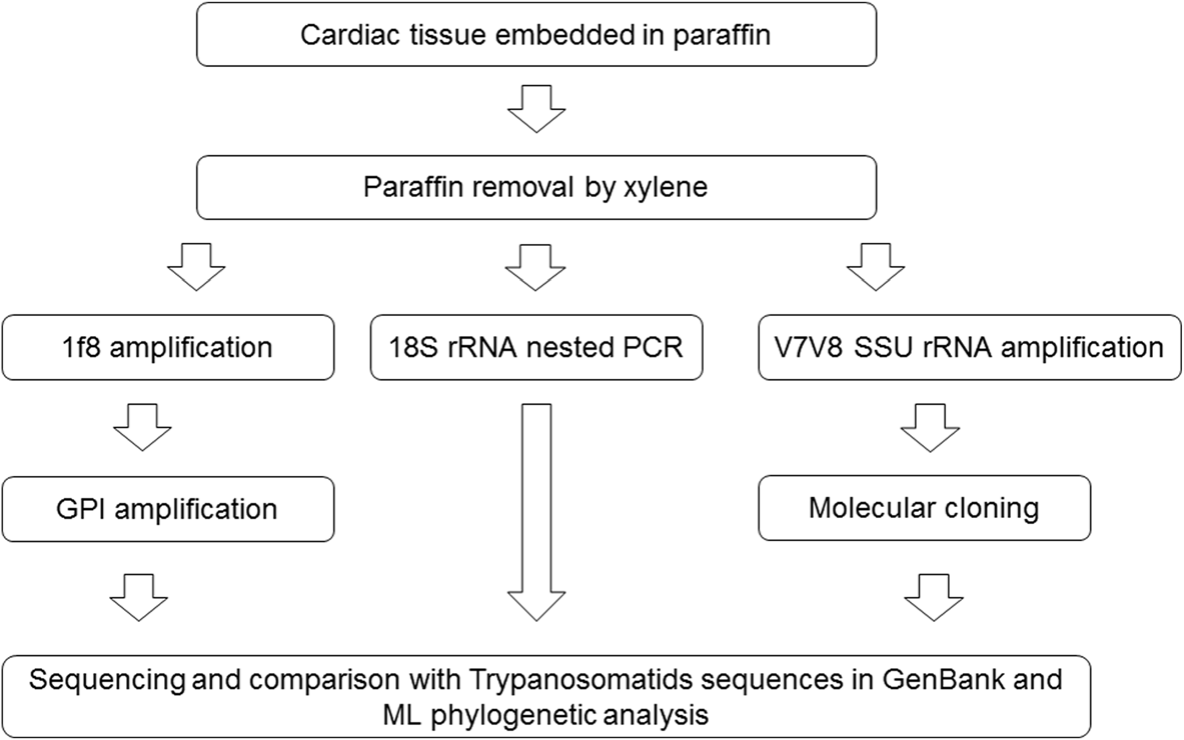
Algorithm of the methodology used for *Trypanosoma cruzi* DTU genotyping in cardiac tissue embedded in paraffin

All of the samples were sequenced for both strands of DNA with the BigDye Terminator v3.1 Cycle Sequencing Kit (Applied Biosystems, Foster city, CA, USA) on an ABI 3730 DNA sequencer available on the PDTIS/FIOCRUZ sequencing platform (Fig. 2). Two clones generated poor sequences and were excluded from the analysis.

#### Sequence and phylogenetic analysis

The sequences were edited, aligned and corrected using the BioEdit software. The sequences were compared with nucleotide sequences deposited in GenBank using the BLAST (Basic Local Alignment Search Tool) algorithm. Phylogenetic tree construction was performed using Mega 5 software [50]. We used the maximum likelihood (ML) method, employing the best DNA model. The best substitution model was identified as having the lowest Bayesian Information Criterion  score (BIC): Hasegawa-Kishino-Yano for the 1f8 gene, Tamura 3+ G (a gamma-distributed rate of variation among sites) parameter for the GPI gene, Kimura 2-parameter for the 18S rRNA gene, and the Kimura 2 + G parameter for V7V8 SSU rRNA, with bootstrapping at 1000 replicates. We used *T. cruzi* (TcI to TcVI), *T. c. marinkellei*, *T. rangeli* and *T. dionisii* sequences from GenBank as references. All sequences analyzed were deposited in the GenBank database under the accession numbers KR905432–KR905446 for the 18S rRNA marker, KT737478 for GPI and KT983981 for 1f8. The GenBank accession numbers can be viewed in Additional file 1.

## Results

### Identification of *Trypanosoma cruzi* DTUs and *T. dionisii* in cardiac tissue

In this study, we decided to use three nuclear markers to genotype the DNA obtained from the cardiac tissue: 1f8, GPI and 18S rRNA genes. Nuclear markers cluster separately with *T. cruzi* DTUs (TcI to TcIV) [44, 51–53]. For the 18S rRNA gene, which exists as thousands of copies in the genome, we used two different regions for characterization: a variable region (V7V8) and a third portion of this variable region [45, 48]. 18S rRNA gene allows identification of different species within the subgenus *Schizotrypanum* and is considered a reliable marker to distinguish between *T. cruzi* DTUs [45, 54–57]. The 1f8 gene allows discrimination between the DTUs TcI and TcIV [44, 46]. The GPI gene, which is also considered a suitable target to distinguish between DTUs, revealed that TcI, TcII, TcIII and TcIV are robust monophyletic groups [5, 13, 52, 58, 59].

We demonstrated via a PCR method the occurrence of four sympatric *T. cruzi* DTUs (TcI, TcII, TcIII and TcIV) in the cardiac tissue of a patient who died in the acute phase of Chagas disease. This is the first time that we observed such a diversity of DTUs in a human case. Furthermore, we also detected *T. dionisii*, a *Trypanosoma* species that has only been described in bats until now, by phylogenetic and additional alignment analyses (Additional file 2).

**Fig. 3:**
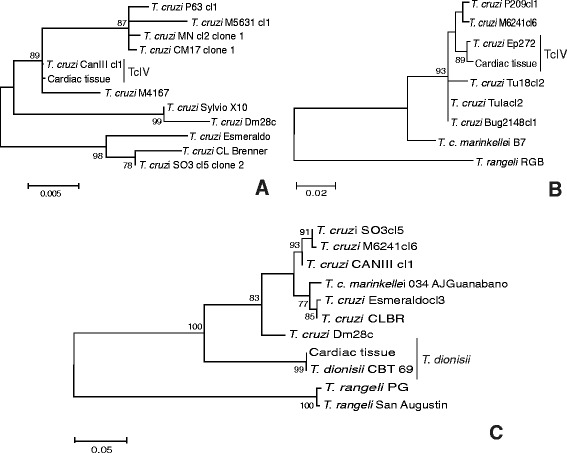
Molecular phylogenetic analysis by maximum likelihood methods. **a** for 1f8 gene with HKY parameter and bootstrap value of 1000 replicates; **b** for GPI gene with T92 + G parameter and bootstrap value of 1000 replicates; and **c** for 18S rRNA K2 parameter and bootstrap value of 1000 replicates. The 1f8 and GPI DNA sequences clustered with DTU TcIV reference strains CANIII (A) and Ep272 (B), and 18S rDNA sequence clustered together with *T. dionisii* isolates. *T. rangeli* isolates RGB, PG and San Augustin were used as the outgroup, except for 1f8 gene, because no sequence is available on GenBank

**Fig. 4:**
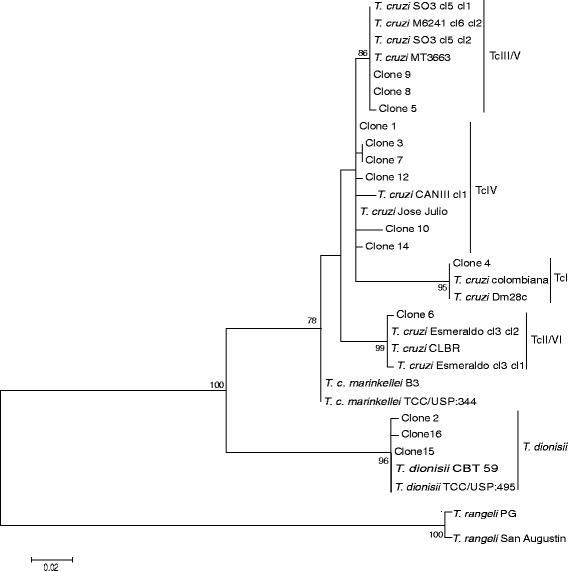
Molecular phylogenetic analysis by maximum likelihood of the V7V8 SSU rRNA gene. K2 + G parameter and bootstrap value of 1000 replicates. The clones from cardiac tissue clustered with Jose Julio and CANIII strains (TcIV); SO3cl5 clone1 and clone2, M6241cl6 clone2 and MT3663 strains (TcIII/TcV); Dm28c and Colombiana strains (TcI); Esmeraldocl3 clone1 and clone2, and CLBR (TcII/TcVI); and *T. dionisii* isolates TCC/USP:495 and CBT 59. *T. rangeli* isolates PG and San Agustin were used as the outgroup

The DTU TcIV was detected in cardiac tissue by employing 1f8 and GPI as molecular targets. The sequence obtained by amplification of the 1f8 gene was subjected to BLAST algorithm and shown to be similar to both TcIII (CM17 and M5631) and TcIV (CANIIII and M4167) strains (96–97 %). According to the ML phylogenetic analysis, the DNA sequence clustered together with the TcIV reference strain CANIII (Fig. 3a). To confirm this result, we sought to determine the occurrence of this DTU based on GPI. With this target, the nucleotide sequence showed 99 % identity to TcIV (Ep272, Saimiri3cl1 and CANIII) strains. In addition, the phylogenetic analysis clustered the sequence with the TcIV reference strain Ep272 (Fig. 3b). DNA from the cardiac tissue was subjected to PCR and molecular cloning of the V7V8 SSU rRNA region. The BLAST algorithm showed that one clone presented a similarity of 100 % to TcI (Dm28c strain), one clone presented a similarity of 99 % to TcII/VI (isolate TCC873, Tulahuencl2, TCC2558), three clones presented similarities of 99 % and 100 % to TcIII (MT3663 strain, isolates TryCC1356 and 1078) and six clones presented similarities of 99–100 % to TcIV (Jose Julio, MT4167 and CanIII strains). The phylogenetic analysis clustered the clones with TcI (*n* = 01), TcII/TcVI (*n* = 01), TcIII (*n* = 03) and TcIV (*n* = 06) (Fig. 4).

Furthermore, three clones were identified as *T. dionisii* via phylogenetic analysis using the ML method. The identification of *T. dionisii* also occurred via sequence analysis of the 18S rRNA gene. The sequence obtained from 18S rRNA nested-PCR was subjected to BLAST algorithm. This sequence showed 100 % identity with *T. dionisii* isolates CBT 63, 64 and 69. In the phylogenetic analysis, the sequence clustered with *T. dionisii* isolates, confirming the presence of *T. dionisii* in this human cardiac tissue sample (Figs. 3c and 4).

### Small mammal capture and *Trypanosoma cruzi* infection

The study area clearly had a reduced mammalian fauna density and diversity. Despite an extensive capture effort involving 840 traps for five nights, only one species of Rodentia (*Trinomys paratus* (n = 5)) and four species of Didelphimorphia [*Didelphis aurita* (n = 1), *Philander frenatus* (n = 1), *Metachirus nudicaudatus* (n = 2) and *Marmosops incanus* (n = 2)] were captured. Additionally, we examined four synanthropic rodents (*Rattus rattus*) that were collected from the peridomicile area. The relative abundance of mammals captured was higher for Didelphimorphia, which represented 54.5 % of the mammals captured, whereas the percentage of Rodentia represented 45.5 %.

None of the sylvatic animals had parasites based on the examination of fresh blood or hemoculture. In serological tests, only three specimens of *T. paratus*, two from Baia Nova and one from Buenos Aires locations, were found to be infected with *T. cruzi* (Fig. 1). The three positive rodents presented only borderline serological titers (1:20) (Table 1).

### Dogs and *Trypanosoma cruzi* infection

Dogs are considered sentinel hosts [60], signaling that *T. cruzi* cycle is occurring in a peridomicile area. Fifteen dogs were examined from the following locations: Rio da Prata -house of the infected patient (n = 10), Baia Nova (n = 2) and Santa Rita (n = 3). Of this total, only two, one from Baia Nova and the other from Santa Rita, displayed only borderline IFAT tests for *T. cruzi* in IFAT (both 1:40) and ELISA (Table 1). All of the dogs (n = 10) from the house where the patient lived were negative for *T. cruzi* based on serological and parasitological tests. This finding means that the dogs from Rio de Prata have not been exposed to *T. cruzi* infection.

**Table 1:** Serological survey in sylvatic and domestic mammals examined in rural areas of the Guarapari municipality

Mammal species	Family	Location	IFAT	ELISA
*Marmosops incanus*	Didelphidae	Baia Nova	1:10	NP
*Marmosops incanus*	Didelphidae	Baia Nova	Negative	NP
*Didelphis aurita*	Didelphidae	Baia Nova	Negative	NP
*Trinomys paratus* (*n* = 2)	Echimyidae	Baia Nova	1:20	NP
*Trinomys paratus*	Echimyidae	Baia Nova	1:10	NP
*Trinomys paratus*	Echimyidae	Baia Nova	Negative	NP
*Metachirus nudicaudatus*	Didelphidae	Buenos Aires	Negative	NP
*Trinomys paratus*	Echimyidae	Buenos Aires	1:20	NP
*Rattus rattus*	Muridae	Santa Rita	Negative	NP
*Metachirus nudicaudatus*	Didelphidae	Todos os Santos	Negative	NP
*Philander frenata*	Didelphidae	Todos os Santos	1:40	NP
*Rattus rattus* (*n* = 2)	Muridae	Todos os Santos	1:10	NP
*Rattus rattus*	Muridae	Todos os Santos	Negative	NP
*Canis familiaris*	Canidae	Baia Nova	1:20	Negative
*Canis familiaris*	Canidae	Baia Nova	1:40	Positive
*Canis familiaris* (*n* = 8)	Canidae	Rio da Prata	1:20	Negative
*Canis familiaris*	Canidae	Rio da Prata	Negative	Negative
*Canis familiaris*	Canidae	Santa Rita	1:20	Negative
*Canis familiaris*	Canidae	Santa Rita	1:40	Positive
*Canis familiaris*	Canidae	Santa Rita	1:40	Negative

*Abbreviations*: *NP* not performed, *IFAT* indirect immunofluorescence antibody test, *ELISA* Enzyme-Linked Immunosorbent Assay

Rodents with serological titles above 1:10 and marsupials and dogs with serological titles above 1:40 were considered positive

### Triatomine infection and molecular characterization

Five triatomine specimens were delivered to the ZCC during our expedition. All of the specimens were identified as *T. vitticeps*. These specimens were from São Miguel (*n* = 1), Rio da Prata (*n* = 2) and Baia Nova (*n* = 2). Four *T. vitticeps* (75 %) had *T. cruzi* based on the intestinal content exam. Only one sample from Rio da Prata (LBT 3214) was negative.

**Fig. 5:**
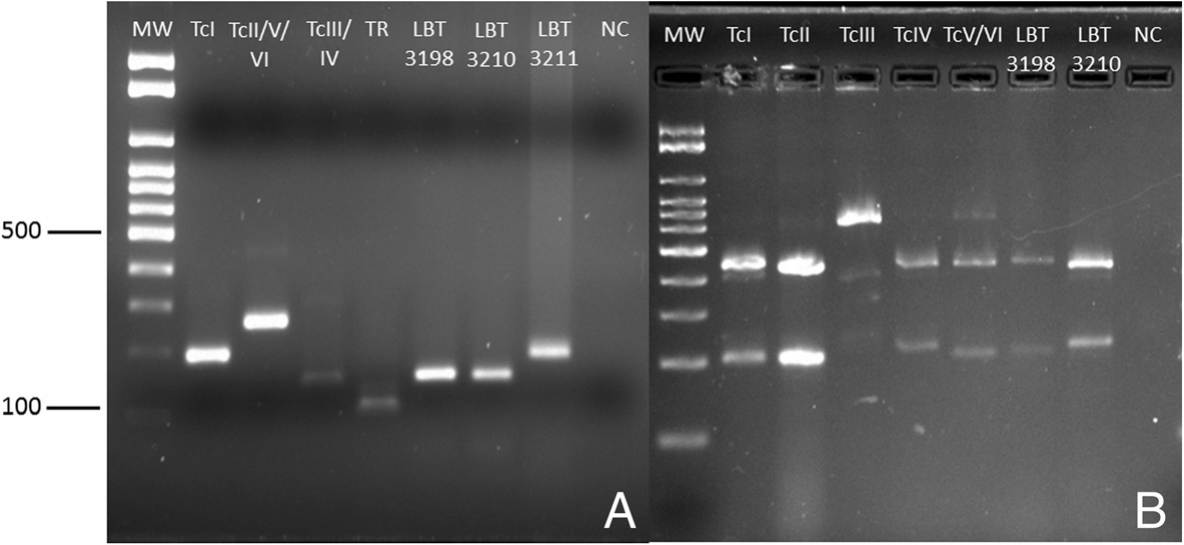
*Trypanosoma cruzi* DTU molecular characterization of *T. vitticeps* isolates LBT 3198, LBT 3210 and LBT 3211. **a** Products amplified by the mini-exon gene and analyzed by electrophoresis on 2 % agarose gel stained with ethidium bromide. The sample LBT 3198 and LBT 3210 presented an amplicon size of 150 bp and was identified as zimodeme 3 (DTU TcIII-TcIV). The sample LBT 3211 was identified as TcI, as it presented an amplicon size of 200 bp. **b** PCR-RFLP with histone 3 gene/AluI restriction enzyme for *T. cruzi* DTU genotype on 3 % agarose gel stained with ethidium bromide. The samples LBT 3198 and LBT 3210 presented amplicons sizes of 210 bp and 440 bp, genotyping these samples as DTU TcIV. DTU reference strains: TcI-SylvioX/10cl1, TcII-Esmeraldocl3, TcIII-M5631cl5, TcIV-9212102R, TcV/TcVI-SC43cl1, *T. rangeli*-Choco, NC:negative control

Four positive samples were subjected to culture and three samples presented epimastigote forms. Molecular characterization using the non-transcribed spacer of the mini-exon gene was performed, which classified the samples as TcI (DTU TcI) - LBT 3211 and Z3 (DTU TcIII/TcIV) - LBT 3198 and LBT 3210 (Fig. 5a). To discriminate between TcIII and TcIV, which is not possible with the mini-exon gene, the LBT 3198 and LBT 3210 samples were further characterized at the DTU level using the H3 marker, resulting in their classification as TcIV (Fig. 5b).

## Discussion

Another fatal ACD case acquired via the oral route has been reported. Studies investigating this mechanism have attracted attention due to the high number of cases and outbreaks in Brazil, especially in the Amazon region, in addition to other South American countries [61–63]. This new/ancient epidemiological profile of the disease must be studied from a novel perspective because the control measures that are used for the elimination of *T. infestans* (domiciliary vector) are not well suited for the current threat. Moreover, all rural locations in Guarapari municipality are now at risk of experiencing CD because residents continue to observe triatomine invasions in their residences and, further, are not aware of the oral route of transmission of *T. cruzi*.

In this study, valuable information was collected regarding ACD due to the unfortunate death of a young patient. Indeed, based on the direct evaluation of infected tissue, a mixed infection by four *T. cruzi* DTUs (TcI, TcII, TcIII and TcIV) was detected in a concomitant infection with *T. dionisii*, a bat trypanosomatid. Fixed biological material, such as tissue embedded in paraffin, for diagnoses is an important source to investigate and understand epidemiology [64]. DNA recovered from this type of material is well maintained and does not result in non-specific bands [65–67]. Moreover, *T. cruzi* has already been diagnosed from mummies [68–70], whose tissue is highly degraded.

This is the first report of a mixed *T. cruzi* infection by four DTUs, identified using DNA extracted directly from human cardiac tissue. Mixed *T. cruzi* DTU infections have been described in several different mammal and triatomine species. Cura et al. [71] reported mixed infections by TcI-IV, TcI-III/IV and TcIII-TcIV in different triatomine species on the American continent. In the Amazon region, Lima et al. [72] reported that *R. pictipes* exhibited a mixed infection of TcI and TcII. Concomitant infections (TcI-TcII and TcII-TcIV) were detected in tissue samples of rodents from the USA [73]. Mixed infections by two or three DTUs in free-living wild mammals have also been described [74]. In humans, mixed infections by two or three DTUs in chronically infected patients have been described in Colombia, Argentina, Chile and in Bolivian patients’ residing in Spain [75–79].

*T. vitticeps* specimens exhibiting mixed infections with TcI-IV, TcII-III-IV and TcI-TcII in ES have previously been observed by our group (Dario, unpublished data). In the present study, observations were conducted in Guarapari, a municipality of ES, where we observed mixed infections of *T. vitticeps* by TcI and TcIV. The finding of simultaneous infection by four *T. cruzi* DTUs in cardiac tissue is consistent with the genotype diversity observed in the state. A high diversity of *T. cruzi* DTUs is not usually observed in other regions in Brazil: in Poço das Antas (Rio de Janeiro state), where TcII is the main DTU infecting monkeys, TcI infection is rare; in Piauí state, TcI and a few cases of TcII infection have been reported; in Santa Catarina state, both TcI and TcII were reported; and in Pará state, TcI infection has been reported [21, 37, 80–82]. In addition, mixed DTU infections in *T. vitticeps* may be attributable to differentially infected blood meal sources or mixed DTU infections in mammals.

The DTUs that we detected infecting the patient have already been described in human infections in addition to presenting a large host range. TcI is the most widespread DTU in nature and is primarily responsible for human infections in the Amazon basin in Brazil, Colombia and Venezuela [11, 16, 83]. TcII, which was classically associated with the Southern cone of South America [10, 84], has already been found in the Brazilian Amazon basin, Colombia, Mexico and USA [15, 72, 73, 85, 86]. In nature, TcIII, which was classically associated with the terrestrial transmission cycle and the armadillo from *Dasypus novemcinctus*, has previously been identified infecting dogs, rodents and marsupials [5, 74, 87–89]. TcIV has also demonstrated a much larger host range as this DTU has been isolated from primates, coati, marsupial, bats, rodent species and *Rhodnius brethesi* triatomines [73, 74, 90]. This study has shown for the first time that TcIII and TcIV are related to human infection in ES. Until now, these DTUs have only been reported in the Amazon—TcIII and TcIV [12, 13], in Bolivian patients in Spain-TcIV [79], in the Southern and Northeast parts of Brazil—TcIII [91, 92], in Minas Gerais state-TcIII [93] and in Argentina-TcIII [94]. These findings show that the distribution of TcIII and in particular TcIV is higher than has been assumed up to now and confirm that these two DTUs are involved in human infection. Moreover, this finding warns of the danger of establishing associations between a parasite species or a parasite genotype and pathogenicity, course of infection or epidemiology. Indeed, a disease is the result of the interaction of several variables, including the peculiarities of a host specimen.

For the first time, the presence of *T. dionisii* has been observed in a human sample. This species, which is closely related to *T. cruzi*, is able to invade mammalian cells as previously demonstrated experimentally [95, 96] and to form cysts in cardiac tissue [97]. We detected *T. dionisii* directly from cardiac tissue more than two weeks after the infection of the patient. This finding indicates that we demonstrated that *T. dionisii* is able to invade and differentiate in human cells. Bat trypanosomatid infections are likely self-resolving and we hypothesize that we would not have been able to detect the parasite at later stages of infection. *T. dionisii* is widely distributed in ES and has been reported in the northern part of the state (Pinheiros municipality) in the bat species *Sturnira lillium*, *Carollia perspicillata*, *Desmodus rotundus*, *Myotis nigricans* and *Lophostoma brasiliensis* [98], and particularly in Guarapari, in bats of *Carollia* species (Dario, unpublished data). The vector of *T. dionisii* is still unknown. There has only been one report on Cimicid insects that maintained an experimental infection by *T. dionisii* [99].

Monogenetic and non-human digenetic trypanosomatid species have already been described to infect humans. Trypanosomes from the subgenus *Herpetosoma* (*T. lewisi*, *T. lewisi*-like), subgenus *Dutonella* (*T. vivax*), subgenus *Trypanozoon* (*T. b. brucei* and *T. evansi*), subgenus *Nannomonas* (*T. congolense*) and the genus *Leptomonas* (*Leptomonas seymouri*) were identified as infecting humans in Africa and Asia [100–103]. *Leishmania tarentolae*, a species that typically occurs in lizards, has been identified in mummies [104]. Additionally, TcBat, a *T. cruzi* DTU reported in bats from different Latin American countries [8, 56, 105], has been described in mummies [71] and in a child from Colombia [106]. These findings show that trypanosomatids are biologically plastic and may be host generalist parasites.

Parasite maintenance via cultivation in axenic media or by passaging in experimentally infected animals results in selective pressure [17, 79, 107], making it difficult to detect the assemblage of clonal components of a given *T. cruzi* isolate. Additionally, during the course of infection, the infected host exerts selective pressures on the parasite population. As a result, during the course of the infection or due to differences in the growth rates of specific populations [108, 109], some populations may be preferentially selected over others [107]. Our study reinforces the importance of the direct characterization of biological samples. In this case, which was a case of CD acquired by the oral route; it is possible that the identification of all of the *T. cruzi* DTUs and *T. dionisii* would not have been possible if we had analyzed the hemoculture of the patient or the same cardiac tissue in the chronic phase of the disease.

In nature, the detection of several different parasite species in the same host is common. Furthermore, the impact of mixed infections on a host is still not well understood. Numerous models and experimental studies have been carried out and in general, they have concluded that mixed infections can affect the host immune response and result in increases in virulence [110, 111]. Female tamarins infected with *T. cruzi* and Acanthocephala (intestinal helminths) may experience increases in the rates of *T. cruzi* infection [112, 113]. Araujo et al. [114] observed that isolated TcI grew faster under culture conditions than Tcl in mixed infections with TcII. Single infections, if they occur in nature, are rare [115, 116]. However, the complexity of this phenomenon means that there are several aspects that still require clarification. Mixed infections may occur after serial exposure to different genotypes and species of parasites at different time intervals and by distinct routes. Here, we know for sure that the patient was infected by the oral route with the four *T. cruzi* genotypes and *T. dionisii* on the same occasion. The fact that *T. cruzi* and *T. dionisii* are within the subgenus *Schyzotrypanum* and were found to occupy the same habitat leads to the hypothesis that an increase in virulence and pathogenicity may occur through competition for resources or alterations in doubling time or impairment in immune clearance, or through a combination of all of these factors.

We observed that the Atlantic rainforest remnants in the study area are suffering from degradation, as demonstrated by the low capture success (1.3 %) during the fieldwork. However, despite degradation, six different mammal species were captured, indicating that the area contains a moderate diversity of small wild mammal species. It has been reported that *T. vitticeps* presents high *T. cruzi* infection rates (more than 60 % of the triatomines are infected) [30, 117, 118]. The absence of positive hemocultures and borderline serological titers showed that the animals examined presented low force of infection and strongly suggests that triatomines are not being infected in the peridomicile area but in distant forest fragments. In this case, the capacity for flight in *T. vitticeps* may be much higher than reported for other insects of this genus. Nothing is known concerning the flight capacity of *T. vitticeps. T. infestans* is capable of flying 200 m or more [119, 120] because it is capable of flying with wind assistance [121]. Another explanation for the high *T. cruzi* infection rates in *T. vitticeps* is that other non-sampled mammals, such as armadillos and bats, can be responsible for parasite maintenance [37, 87, 89, 122–124].

## Conclusion

In conclusion, our results indicate that (i) mixed infections in humans may be more common than has been recognized up to now and should be taken into consideration in future studies; (ii) the distribution of *T. cruzi* TcIII and TcIV in Brazilian biomes is broader than has been assumed until now, and the putative associations between *T. cruzi* DTUs and host species, geographical distribution and pathogenicity still pose epidemiological challenges; and (iii) *T. dionisii* is able, at least, to colonize human heart muscle cells.

## Additional files

Additional file 1: Table S1. *Trypanosoma cruzi* and *Trypanosoma dionisii* GenBank accession numbers for the 1f8, GPI and 18S rRNA genes. (DOCX 13 kb) Additional file 2: Table S2. Alignment sequences from *Trypanosoma cruzi*, *Trypanosoma cruzi marinkellei*, *Trypanosoma dionisii* species isolates, and V7V8 SSU rRNA clones obtained from cardiac tissue. The dots are representing same base position for *T. cruzi*. The stars are representing same base position for *T. dionisii*. (*DOCX 17 kb*)

## Abbreviation

ACD: Acute chagas disease
BLAST: Basic local alignment search tool
CD: Chagas disease
COLTRYP: Coleção de trypanosoma de mamíferos silvestres, domésticos e vetores
DTU: Discrete typing unit
ELISA: Enzyme-Linked Immunosorbent Assay
ES: Espírito Santo state
GPI: Glucose-phosphate isomerase
IFAT: Indirect Immunofluorescent Antibody Test
LIT: Liver Infusion Tryptose
ML: Maximum likelihood
NNN: Novy Mc Neal Nicole
PCR: Polymerase chain reaction
RFLP: Restriction fragment length polymorphism
Sesa/ES: Espírito Santo state Health Department
ZCC: Zoonosis Control Center
