# Supplementary material for: Ecological scenario and Trypanosoma cruzi DTU characterization of a fatal acute Chagas disease case transmitted orally (Espírito Santo state, Brazil)
Source: Parasit Vectors. 2016 Aug 31;9(1):477. doi: 10.1186/s13071-016-1754-4 (PMC5006519; doi:10.1186/s13071-016-1754-4)
Supplement: Additional file 1: — Table S1. Trypanosoma cruzi and Trypanosoma dionisii GenBank accession numbers for the 1f8, GPI and 18S rRNA genes. (DOCX 13 kb) [file 13071_2016_1754_MOESM1_ESM.docx]

Additional file 1 *Trypanosoma cruzi* and *Trypanosoma dionisii* GenBank accession numbers for the 1f8, GPI and 18SrRNA genes.

| Sample | Gene | Molecular characterization | GenBank accession number |
| --- | --- | --- | --- |
| Cardiac tissue | 1f8 | DTU TcIV | KT983981 |
| Cardiac tissue | GPI | DTU TcIV | KT737478 |
| Cardiac tissue | 18S rRNA | *T. dionisii* | KR905432 |
| Clone 1 | V7V8 SSU rRNA | DTU TcIV | KR905433 |
| Clone 2 | V7V8 SSU rRNA | *T. dionisii* | KR905444 |
| Clone 3 | V7V8 SSU rRNA | DTU TcIV | KR905434 |
| Clone 4 | V7V8 SSU rRNA | DTU TcI | KR905435 |
| Clone 5 | V7V8 SSU rRNA | DTU TcIII | KR905436 |
| Clone 6 | V7V8 SSU rRNA | DTU TcII | KR905437 |
| Clone 7 | V7V8 SSU rRNA | DTU TcIV | KR905438 |
| Clone 8 | V7V8 SSU rRNA | DTU TcIII | KR905439 |
| Clone 9 | V7V8 SSU rRNA | DTU TcIII | KR905440 |
| Clone 10 | V7V8 SSU rRNA | DTU TcIV | KR905441 |
| Clone 12 | V7V8 SSU rRNA | DTU TcIV | KR905442 |
| Clone 14 | V7V8 SSU rRNA | DTU TcIV | KR905443 |
| Clone 15 | V7V8 SSU rRNA | *T. dionisii* | KR905445 |
| Clone 16 | V7V8 SSU rRNA | *T. dionisii* | KR905446 |
