# Supplementary material for: Ecological scenario and Trypanosoma cruzi DTU characterization of a fatal acute Chagas disease case transmitted orally (Espírito Santo state, Brazil)
Source: Parasit Vectors. 2016 Aug 31;9(1):477. doi: 10.1186/s13071-016-1754-4 (PMC5006519; doi:10.1186/s13071-016-1754-4)
Supplement: Additional file 2: — Table S2. Alignment sequences from Trypanosoma cruzi, Trypanosoma cruzi marinkellei, Trypanosoma dionisii species isolates, and V7V8 SSU rRNA clones obtained from cardiac tissue. The dots are representing same base position for T. cruzi. The stars are representing same base position for T. dionisii. (DOCX 17 kb) [file 13071_2016_1754_MOESM2_ESM.docx]

Additional file 2 Alignment sequences from *Trypanosoma cruzi*, *Trypanosoma cruzi marinkellei*, *Trypanosoma dionisii* species isolates, and V7V8 SSU rRNA clones obtained from cardiac tissue. The dots are representing same base position for *T. cruzi*. The stars are representing same base position for *T. dionisii*.

| Isolate/clone | Nucleotide position | | | | | | | | | | |
| --- | --- | --- | --- | --- | --- | --- | --- | --- | --- | --- | --- |
|  | 190 | 200 | 221 | 316 | 393-401 | 414-415 | 430-432 | 448 | 465 | 481-483 | 494 |
| *T. cruzi* Dm28c | T | A | A | C | TTATTCCA | TT | TGG | T | A | GCA | T |
| *T. cruzi* Y | **.** | **.** | **.** | **.** | **. . . . . . . .** | **. .** | **. . .** | **.** | **.** | **. . .** | **.** |
| *T. cruzi* 3663 | **.** | **.** | **.** | **.** | **. . . . . . . .** | **. .** | **. . .** | **.** | **.** | **. . .** | **.** |
| *T. cruzi* CANIII cl1 | **.** | **.** | **.** | **.** | **. . . . . . . .** | **. .** | **. . .** | **.** | **.** | **. . .** | **.** |
| *T. cruzi* SO3 cl5 clone 2 | **.** | **.** | **.** | **.** | **. . . . . . . .** | **. .** | **. . .** | **.** | **.** | **. . .** | **.** |
| *T. cruzi* TCC/USP: 499 | **.** | **.** | **.** | **.** | **. . . . . . . .** | **. .** | **. . .** | **.** | **.** | **. . .** | **.** |
| Clone 1 | **.** | **.** | **.** | **.** | **. . . . . . . .** | **. .** | **. . .** | **.** | **.** | **. . .** | **.** |
| Clone 3 | **.** | **.** | **.** | **.** | **. . . . . . . .** | **. .** | **. . .** | **.** | **.** | **. . .** | **.** |
| Clone 4 | **.** | **.** | **.** | **.** | **. . . . . . . .** | **. .** | **. . .** | **.** | **.** | **. . .** | **.** |
| Clone 5 | **.** | **.** | **.** | **.** | **. . . . . . . .** | **. .** | **. . .** | **.** | **.** | **. . .** | **.** |
| Clone 6 | **.** | **.** | **.** | **.** | **. . . . . . . .** | **. .** | **. . .** | **.** | **.** | **. . .** | **.** |
| Clone 7 | **.** | **.** | **.** | **.** | **. . . . . . . .** | **. .** | **. . .** | **.** | **.** | **. . .** | **.** |
| Clone 8 | **.** | **.** | **.** | **.** | **. . . . . . . .** | **. .** | **. . .** | **.** | **.** | **. . .** | **.** |
| Clone 9 | **.** | **.** | **.** | **.** | **. . . . . . . .** | **. .** | **. . .** | **.** | **.** | **. . .** | **.** |
| Clone 10 | **.** | **.** | **.** | **.** | **. . . . . . . .** | **. .** | **. . .** | **.** | **.** | **. . .** | **.** |
| Clone 12 | **.** | **.** | **.** | **.** | **. . . . . . . .** | **. .** | **. . .** | **.** | **.** | **. . .** | **.** |
| Clone 14 | **.** | **.** | **.** | **.** | **. . . . . . . .** | **. .** | **. . .** | **.** | **.** | **. . .** | **.** |
| *T. c. marinkellei* TryCC 1093 | **.** | **.** | **.** | A | **. . . . . . . .** | - - | **. . .** | **.** | **.** | **. . .** | **.** |
| *T. dionisii* TCC/USP: 495 | C | T | G | A | ATGATATC | CA | GCA | G | G | ACG | C |
| Clone 2 | ***** | ***** | ***** | ***** | * * * * * * * * | * * | * * * | * | * | * * * | * |
| Clone 15 | ***** | ***** | ***** | ***** | * * * * * * * * | * * | * * * | * | * | * * * | * |
| Clone 16 | ***** | ***** | ***** | ***** | * * * * * * * * | * * | * * * | * | * | * * * | * |

- - Gap position
